# Treatment outcomes of tuberculosis cases by HIV status in Haramaya General Hospital, Ethiopia: A retrospective cross-sectional study

**DOI:** 10.1097/MD.0000000000038034

**Published:** 2024-05-03

**Authors:** Adnan Ahmed, Fitsum Weldegebreal, Fikru Tebeje, Yadeta Dessie

**Affiliations:** a Eastern Harargi Health Bureau, Oromia, Ethiopia; b School Medical Laboratory Sciences, College of Health and Medical Sciences, Haramaya University, Harar, Ethiopia; c School of Public, College of Health and Medical Sciences, Haramaya University, Harar, Ethiopia.

**Keywords:** coinfection, eastern Ethiopia, human immunodeficiency virus, treatment outcomes, uberculosis

## Abstract

Tuberculosis (TB) and human immunodeficiency virus (HIV) coinfection pose significant challenges to global health, particularly in achieving the target of ending TB. However, the impact of HIV status on TB treatment outcomes remains unclear, especially in eastern Ethiopia. This study aimed to assess the treatment outcomes of TB cases by HIV status and associated factors in Haramaya General Hospital from November 15 to December 30, 2022. A retrospective cross-sectional study was conducted, reviewing the TB registry and treatment cards of patients who received anti-TB treatment between September 2017 and August 2022. Of the 420 samples addressed, 91.0% (95% CI: 88.3%–96.7%) of all TB patients had successful treatment outcomes. The treatment success rates of HIV-positive and HIV-negative TB patients were 80.0% and 91.9%, respectively. Being HIV-negative (AOR: 2.561, 95% CI: 1.002–6.542), being in the age group of 20 to 35 years (AOR: 2.950, 95% CI: 1.171–7.431), and urban residence (AOR: 2.961, 95% CI: 1.466–5.981) were associated with the TB treatment success rate. There was a high treatment success rate among all patients with TB. HIV status was associated with TB treatment outcomes. Strengthening TB-HIV collaborative activities, providing patient-centered care and support, and frequent monitoring and evaluation are recommended to improve the TB success rate.

## 1. Introduction

Globally, in 2017, there were an estimated 10.0 million new cases of tuberculosis (TB). Among these TB cases, 1 in 11 were people living with HIV (PLHIV). In the same year, out of 1.6 million deaths of TB cases worldwide, almost 1 in 5 was due to HIV coinfection. Of the estimated 9% of people who were HIV-positive TB patients, almost 3 (72%) were in the WHO African region. The number of HIV-infected TB cases is the highest in Africa.^[1]^ Ethiopia is 1 of the 30 high TB and TB/HIV coinfected burden countries globally, which contributes to 84% of TB cases and 83% cases of HIV-positive TB cases as the WHO estimated from 2016 to 2020.^[2]^ In 2018, among the newly registered people for TB treatment, treatment success rates were 85% and 76% for TB patients who live with HIV, respectively.^[3]^

Worldwide, in 2019, approximately 1-in-7 (208,000/1,418,000) of all TB-related deaths are associated with HIV. Among these deaths, 4 in 5 (169,000/208,000) HIV-infected TB cases are in Africa. Of the 2.5 million people with TB in Africa, 1 in 4 (0.6 million) is infected with HIV.^[3]^ Major improvements in global TB control followed the extensive implementation of DOTS which is the most curative method for TB, though the high mortality rate of HIV-infected TB patients continues. WHO recommends antiretroviral therapy (ART) to save the lives of TB patients with known positive HIV status and all TB patients living in HIV-prevalent settings, though the complexity of treating 2 infections requires multi-drug therapy at the same time.^[4]^

A systematic review and meta-analysis study conducted on TB treatment outcomes and predictors in Africa reported that the overall TB treatment success rate was 79.0%. TB/HIV coinfection was significantly associated with an amplified risk of unsuccessful treatment outcomes compared to HIV-negative TB patients.^[5]^ In contrast to this, a retrospective cross-sectional study conducted on the trends of notification rates and treatment outcomes of TB cases with and without HIV confection in 8 rural districts of Uganda between 2015 and 2019 showed that the overall TB treatment success rate was 72.5.0%. In the same study, the TB treatment success rate was found to be significantly lower among HIV-free TB patients (71.2%) compared to TB/HIV coinfected patients (74.0%), *P* < .001.^[6]^ This study added factors like diabetes mellitus (DM) comorbidity, co-trimoxazole preventive therapy, and ART initiation, besides impact of HIV status, and demographic and TB treatment-related factors, to estimate the treatment outcome of TB in HIV-negative and HIV-positive TB patients.^[7]^

In Ethiopia, according to the Institute for Health Metrics and Evaluation for Ethiopia, TB is the fourth communicable disease and the most common cause of death in 2019.^[8]^ The top 10 reported reasons for death among hospital admissions include TB. TB remains the leading cause of death for PLHIV, accounting for around 40% of AIDS-related deaths, though the national responses to jointly address the TB and HIV epidemics began in 2004 and have succeeded in saving the lives of affected citizens.^[9]^ According to a cross-sectional analytic study conducted on outcomes of TB treatment in HIV coinfected TB patients in Ethiopia, the overall treatment success rate was 91.5% and was significantly higher among HIV-negative cases (93.6%).^[10]^ However, a 5-years retrospective document review conducted at Jimma University Medical Center to estimate the treatment outcomes of TB and associated factors between September 1, 2012, and August 31, 2017 reported that TB/HIV coinfection did not show an association with unsuccessful treatment outcome TB.^[11]^

Different studies identify that old age, female sex, rural residence, diabetic mellitus, pretreatment weight, and years of treatment of TB patients are risk factors for the treatment outcome of TB patients.^[5,12–16]^ Similarly, HIV comorbidity of TB patients, ART initiated, types of therapy, co-trimoxazole preventive therapy, types of TB, and categories in TB patients are factors associated with treatment outcomes of TB patients.^[10,17–22]^

TB is preventable, and understanding risk factors can help identify vulnerable groups and help reduce unsuccessful treatment outcomes.^[11,12,18]^ TB among PLHIV is preventable and curable. To improve the TB success rate, WHO recommends appropriate treatment of TB, joint, TB and HIV programming on collaborative TB/HIV activities, and preventive therapy. Strengthening screening and care for DM undertaken in all settings for all persons with TB is recommended.^[7]^ However, in Ethiopia, as in other resource-constrained countries, such reports are primarily limited, and the contribution of treatment outcomes to TB cases by HIV status is not well documented, and no research has been conducted in eastern Ethiopia. Therefore, this study tried to assess the treatment outcomes of TB cases by HIV status and its associated factors in eastern Ethiopia.

### 1.1. Study area and period

Haramaya General Hospital (HGH), located approximately 510 km from Addis Ababa in the East Hararghe Zone of Oromia Regional State, was selected as the study site based on convenience (geographic accessibility) and the presence of a substantial patient load. HGH has a unique patient demographic and epidemiological profile that makes it an important setting for studying TB cases and treatment outcomes.

Originally established as a health center, HGH was upgraded to a hospital level in 2005 and currently serves as a vital healthcare facility for over 1.2 million people in its catchment areas. The hospital plays a crucial role in providing medical care to a large population, making it an ideal site for studying TB and its related dynamics. Within the hospital, the Directly Observed Treatment, Short-course (DOTs) clinic serves as the primary facility for managing TB cases. Annually, the DOTs clinic admits approximately 107 TB patients, indicating a significant burden of TB in the region. This high patient volume allows for a comprehensive evaluation of TB treatment outcomes and associated factors.^[23]^ In this study, data collection took place from November 15 to December 30, 2022, capturing a specific timeframe of TB cases and their treatment outcomes. By examining the data during this period, the study aims to provide valuable insights into the treatment outcomes of TB cases by HIV status in HGH, shedding light on the local context and contributing to the global understanding of TB-HIV coinfection dynamics.

By focusing on HGH, this study aims to provide a detailed analysis of TB treatment outcomes in a specific setting, considering the unique patient demographics, epidemiology, and healthcare infrastructure of the region.

### 1.2. Study design and population

A facility-based retrospective cross-sectional study design was used. Three hundred eighty TB patients who had been diagnosed with active TB disease and enrolled in a course of anti-TB treatment regimens from September 1, 2017, to August 31, 2022, in the HGH were included. Any TB patient on the second-line TB treatment register was excluded from the study.

### 1.3. Sample size determination and sampling technique

Since this study aimed to compare two proportions of the treatment success rate of HIV-infected TB patients and TB-only patients, the sample size was determined by using the double population proportion formula and by considering the following statistical assumptions: the 2-side CI: 95%, the desired power: 80%, and P = Proportion of the TB treatment success rate among TB patients registered in DOTs clinics of hospitals for anti-TB treatment services. The prevalence of HIV-incident TB cases in Ethiopia is estimated to be 22%.^[24]^ Even though the maximum calculated sample size of the study was 380, all 425 TB patients who fulfilled the inclusion criteria, and were diagnosed with active TB disease and put on anti-TB treatment regimens in the HGH from September 1, 2017, to August 31, 2022, were included.

### 1.4. Data collection tools and procedures

Data were extracted through medical records reviews of TB patients using a structured checklist designed for this study. The contents of the checklist included sociodemographic data (year of treatment, sex, age, residence, pretreatment weight), HIV status, DM, type of TB, TB treatment category, ART, co-trimoxazole given, duration of treatment, and treatment outcomes of active TB patients. The data were collected from the first date of the TB patients registered for first-line TB treatment up to the end of the treatment or interruption.

## 2. Methods of data analysis

Data were checked for completeness every day. Study participants who were on treatment during the data collection period between November 15 and December 30, 2022, were excluded from the study sample in order to reduce negative effects on the study output and data quality. The continuous variables age, pretreatment weight, and distance to the hospital were recorded into groups for the purpose of the analysis. The treatment outcomes were also recoded into 2 groups successful treatment outcome and unsuccessful treatment outcomes. The data were summarized using frequencies and percentages. Any errors identified at this time were corrected after a review of the original data using the code numbers. The proportion for categorical variables was compared using a chi-square test for HIV-negative and HIV-positive TB patients. Bivariate and multiple logistic regression models were used to identify significant associated factors of TB treatment outcome in all TB patients with 95% CIs.

Binary logistic regression was used to calculate the crude odds ratio with a 95% CI. Each explanatory variable was entered into a binary logistic regression model so as to determine the presence of a statistically significant association with the dependent variable. Multicollinearity among selected independent variables was checked through Pearson’s correlation, and only treatment duration was found and excluded from logistic regression analysis. All the explanatory variables that showed a statistically significant association, at least either in a cumulative *P* value or in a categorical group of the variable in the bivariate analysis, were included in the final multivariate logistic model in order to identify the significantly independent factors of TB treatment outcome. The criterion for significance was set at *P* < .05 and at 95% confidence interval (CI).

### 2.1. Data quality control

Data quality was ensured by carefully designing data extraction tools. Pretesting of data collection tools was done in the DOTS clinic of the hospital on 30 patients’ records, accounting for 7.1% of those started TB treatment services 5 months before the study period within 1 week, with the whole research team after 1 day of training given for both the data collectors and supervisor before the start of the data collection. The overall activities of data extraction were monitored by the researchers and supervisor during data collection. The data collectors were supervised daily by the supervisor, and all filled formats were reviewed by the researchers for completeness during data collection. Finally, all the collected data were checked by the supervisor and investigator for completeness and consistency during the data management, storage, and analysis. Consistency was examined through a random selection of 5% of the sample by the researchers.

### 2.2. Ethical consideration

Ethical clearance was obtained from Haramaya University, College of Health and Medical Science Ethical Review Committee with an ethical reference number (Ref. No. IHRERC/182/2022). Then letters of cooperation were written to HGH and concerned bodies. Permission was obtained from DOTS clinics at the hospital. Following these, searching and obtaining the selected samples’ registers were processed with the assigned person. Finally, precautions were taken from disclosing patients’ data. Since this was a retrospective study, the consent of patients was not obtained directly. However, patient information was handled anonymously, with ensuring confidentiality. The confidentiality of the participants was ensured by excluding names and identifiers from the questioners and, all collected data was coded and entered into the computer.

## 3. Results

### 3.1. Background characteristics of tuberculosis patients

A total of 425 TB patients were registered at the HGH from September 2017 to August 2022. Five (1.1%) (1 HIV-positive and 4 HIV-negative) TB patients were on treatment during the data collection period, so they were not included in the study analysis since their treatment outcomes were not documented. Therefore, the study analysis was done on the data of 420 TB patients.

The age of the participants ranged from 1 to 80 years, with a median of 22 years (IQR: 17–30 yr). About 74.3% of HIV-positive and 82.9% of HIV-negative TB patients were younger than 36 years of age, respectively. The pretreatment weight of the TB patients ranged from 5 to 86 kg, with a mean (±SD) weight of 44.9 (±15.434) kg. There was no evidence of a statistical difference between the 2 categories of patients to year of treatment (*P* = .937), age (*P* = .083), sex (*P* = .953), place of residence (*P* = .416), and pretreatment weight (*P* = .111) (Table 1).

**Table 1:** Background characteristics of TB patients by HIV status in HGH, 2022 (n = 420).

Variables	HIV-positive TB cases	HIV-negative TB cases	*P* value
	n (%)	n (%)	
Age			.083
<20	6 (17.1)	135 (35.1)	
20–35	20 (57.2)	184 (47.8)	
>35	9 (25.7)	66 (17.1)	
Sex			.953
Male	19 (54.3)	211 (54.8)	
Female	16 (45.7)	174 (45.2)	
Residence			.416
Urban	19 (54.3)	236 (61.3)	
Rural	16 (45.7)	149 (38.7)	
Pretreatment weight			.111
<35	2 (5.7)	73 (19.0)	
35–53	24 (68.6)	208 (54.0)	
>53	9 (25.7)	104 (27.0)	

*Significant at *P* < .05.

### 3.2. Tuberculosis cases by human immunodeficiency virus status

**Figure 1.**
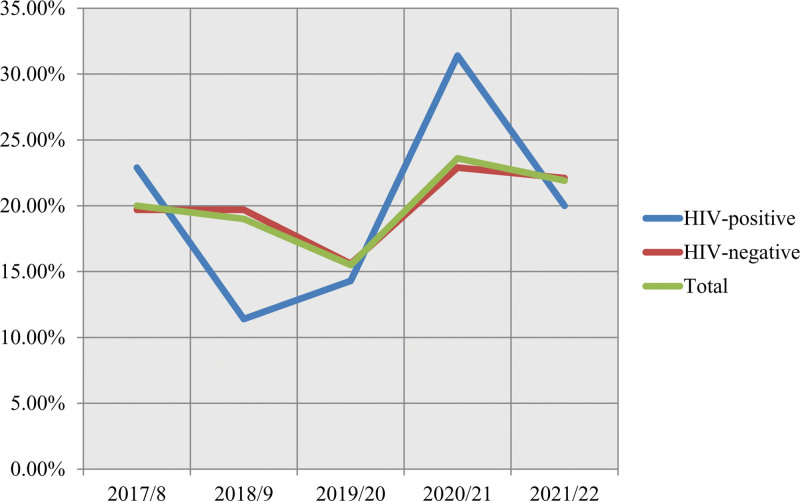
TB cases registered in Haramaya General Hospital by HIV status, September 2017–August 2022.

Linear trends of TB cases by HIV status over the period were explored using chi-square tests. All TB patients knew their HIV status. 8.3% (35/420) (95% CI: 0.057–0.109) of the participants were TB-HIV coinfected patients. There was decreased trend in the proportion of HIV-positive TB cases from 2017/8 to 2019/20, then showed a sharp rise in 2020/21 and displayed a slight decrease from 22.9% to 20.0%. As a result, there was a decreasing trend in the proportion of all TB cases from 2017/8 to 2019/20, which then showed a sharp increase in 2020/21, which finally displayed an increasing TB case trend from 20.0% to 21.9% with a smaller rate related to that of HIV-negative cases (19.7%–22.1%) due to the effect of the HIV-positive rate recorded, though it did not show a statistically significant difference (Fig. 1).

### 3.3. Diseases characteristics of tuberculosis patients

About 95.5% of the patients had no previous history of TB treatment, mostly newly diagnosed cases. About 40.2% of both HIV-positive and HIV-negative TB patients had smear-positive pulmonary TB. There was a statistically significant difference in treatment duration between HIV coinfected and HIV-negative TB patients (*P* = .016). However, there was no statistically significant difference between HIV-negative and HIV-positive TB patients in the previous history of TB treatment (*P* = .204), TB treatment category (*P* = .699), TB type (*P* = .320), and DM (*P* = .469) (Table 2).

**Table 2:** Diseases characteristics of TB patients by HIV status in HGH, 2022 (n = 420).

Variables	HIV-positive TB cases	HIV-negative TB cases	*P* value
	n (%)	n (%)	
TB pre. tr. History			
Yes	3 (8.6)	16 (4.2)	.204
No	32 (91.4)	369 (95.8)	
TB tr. Category			
New TB	31 (88.6)	357 (92.7)	.699
Pre. treated TB	2 (5.7)	15 (3.9)	
Transfer-in	2 (5.7)	13 (3.4)	
TB type			
Smear-positive PTB	10 (28.6)	159 (41.3)	.320
Smear-negative PTB	14 (40.0)	134 (34.8)	
EPTB	11 (31.4)	92 (23.9)	
Treatment duration			.016
<6 mo	7 (20.0)	27 (7.0)	
≥6 mo	28 (80.0)	358 (93.0)	
Diabetes mellitus			.469
Yes	0 (0.0)	3 (0.8)	
No	35 (100)	382 (99.2)	

*Significant at *P* < .05.

**Figure 2.**
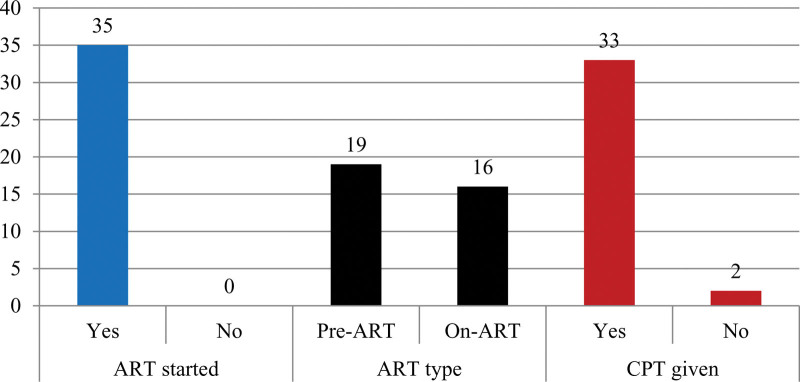
HIV coinfected TB patients with medical care services in Haramaya General Hospital, September 2017–August 2022.

All HIV-positive TB patients started ART services. About 45.7% of HIV-positive TB patients who were already on-ART services while diagnosed with active TB were in the on-ART group, and 54.3% of them were in the pre-ART category. And about 94.3% of HIV-positive TB patients took cotrimoxazole preventive therapy (Fig. 2).

### 3.4. Treatment outcome of tuberculosis patients

The success rate was 91.0% (95% CI: 88.3%–96.7%) for all TB patients. The treatment success rate for HIV-positive TB patients was 80.0% (95% CI: 66.7%–93.3%), and it was 91.9% (95% CI: 89.2%–94.6%) for HIV-negative TB patients. HIV-positive patients had a higher unsuccessful TB treatment outcome of 20%, compared to 8.1% for HIV-negative patients. HIV-positive TB patients were more than twice and twenty-two times more likely to die and lost to follow-up, respectively than HIV-negative TB patients (*P* value = .004) (Table 3).

**Table 3:** Treatment outcomes of TB patients by HIV status in HGH, 2022 (n = 420).

Variables	HIV-positive TB cases	HIV-negative TB cases	Total	*P* value
	n (%)	n (%)		
Treatment outcomes				.004
Cured	9 (25.7)	145 (37.7)	154 (36.7)	
Treatment completed	19 (54.3)	209 (54.3)	228 (54.3)	
Treatment failure	–	1 (0.3)	1 (0.2)	
LTFU	4 (11.4)	2 (0.5)	6 (1.4)	
Died	3 (8.6)	16 (4.2)	19 (4.5)	
Transferred out	–	12 (3.1)	12 (2.9)	
Overall treatment				.028
Successful treatment	28 (80.0)	354 (91.9)	382 (91.0)	
Unsuccessful treatment	7 (20.0)	31 (8.1)	38 (9.0)	

### 3.5. Factors associated with treatment outcome

In bivariate analysis, variables including HIV status, age, and residence were significant at a *P* value less than .05 and considered as a candidate for multivariable analysis. In multivariable analysis; HIV status, age, and residence were significantly associated with TB treatment outcome.

HIV-negative TB patients were 2.561 times more likely to have a successful treatment outcome compared to HIV-positive patients (AOR: 2.561, 95% CI: 1.002–6.542; *P* = .049). Likewise, the odds of having successful treatment outcomes were 2.95 times higher in the age group 20 to 35 years (AOR: 2.950, 95% CI: 1.171–7.431; *P* = .022) as great in TB patients than that of the age group higher than 35 years. Patients who resided in urban area had 2.961 times more likely to have a successful treatment outcome than rural dwellers (AOR: 2.961, 95% CI: 1.466–5.981; *P* = .002) (Table 4).

**Table 4:** Factors associated with treatment success among TB patients in Haramaya General Hospital, 2022 (n = 420).

Variables	Successful treatment	Unsuccessful treatment	*P* value	COR (95% CI)
	n (%)	n (%)		
Year of treatment				
2017/8	73 (19.2)	11 (28.9)		Ref
2018/9	72 (18.9)	8 (21.1)	.148	2.160 (0.762–6.126)
2010/20	61 (16.0)	4 (10.5)	.409	1.593 (0.528–4.803)
2020/21	89 (23.4)	9 (23.7)	.926	0.940 (0.254–3.473)
2021/22	86 (22.6)	6 (15.8)	.498	1.449 (0.495–4.245)
Age (yrs)				
<20	132 (34.6)	9 (23.7)	.608	1.248 (0.535–2.910)
20–35	188 (49.2)	16 (42.1)	.015	3.075 (1.248–7.578)
>35	62 (16.2)	13 (34.2)		Ref
Sex				
Male	210 (55.0)	20 (52.6)		Ref
Female	172 (45.0)	18 (47.4)	.782	0.910 (0.467–1.775)
Residence				
Urban	241 (63.1)	14 (36.8)	.002	2.930 (1.468–5.848)
Rural	141 (36.9)	24 (63.2)		Ref
Pretreatment weight in Kg				
<35	66 (17.3)	9 (23.7)		Ref
35–53	215 (56.3)	17 (44.7)	.769	1.148 (0.458–2.875)
>53	101 (26.4)	12 (31.6)	.304	0.666 (0.306–1.446)
TB pre. tr. History				
Yes	16 (4.2)	3 (7.9)		Ref
No	366 (95.8)	35 (92.1)	.303	1.961 (0.545–7.059)
TB tr. Category				
New TB	355 (92.9)	33 (86.8)		Ref
Pre. treated TB	14 (3.7)	3 (7.9)	.519	0.604 (0.131–2.793)
Transfer-in	13 (3.4)	2 (5.3)	.738	1.393 (0.200–9.711)
TB type				
Smear positive PTB	155 (40.6)	14 (36.8)		Ref
Smear negative PTB	132 (34.6)	16 (42.1)	.879	1.073 (0.434–2.652)
EPTB	95 (24.9)	8 (21.1)	.422	1.439 (0.592–3.501)
Diabetes mellitus				
Yes	2 (0.5)	1 (2.6)		Ref
No	380 (99.5)	37 (97.4)	.231	4.324 (0.432–53.864)
HIV status				
Positive	28 (7.3)	7 (18.4)		Ref
Negative	354 (92.7)	31 (81.6)	.023	2.855 (1.154–7.064)
ART type				
Pre-ART	15 (53.6)	4 (57.1)		Ref
On-ART	13 (46.4)	3 (42.9)	.865	1.156 (0.217–6.145)
CPT given				
Yes	27 (96.4)	6 (85.7)		Ref
No	1 (3.6)	1 (14.3)	.311	0.222 (0.012–4.077)

## 4. Discussion

This study was set to determine treatment outcome of TB by HIV status and its associated factors in the HGH. The overall treatment success for TB patients was 91.0%. About 8.3% of TB patients were HIV-positive and HIV coinfection had poorer TB treatment outcomes. Treatment success rate of HIV-positive TB patients and HIV-negative TB patients were 80.0% and 91.9%, respectively. HIV status, age, and place of residence were significant associated factors for TB treatment success.

There was a high rate of successful treatment outcome (91.0%) for all TB patients. This finding is similar to the findings reported success rate for all TB, HIV-negative and HIV-positive TB patients respectively from Addis Ababa (91.5%, 93.6%, and 88.2%),^[10]^ Dilla (92.3%, 94.9%, and 79.8%),^[25]^ and Debre Tabor (90.1%, 90.8%, and 88.1%).^[26]^ However it is higher than that reported success rate for all TB, HIV-negative and HIV-positive TB patients respectively from Gondar (60.1%, 68.6%, and 49.5%),^[27]^ Northeast Ethiopia (80.7%, 84.5%, and 64%),^[21]^ and Jinka (74%, 78.9%, and 70.6%).^[19]^ On the other hand, our finding is in contrast with a study in 8 rural districts of Uganda reported higher TB success rate 81.9% among HIV coinfected patients compared to 63.9% for HIV-negative TB patients in 2019,^[6]^ and several studies reported no association between TB treatment outcomes and HIV coinfection from Ghana,^[28]^ Jimma University Medical Center,^[11]^ and Northwest Ethiopia.^[26]^ These observed variations might be due to the differences in the quality of TB treatment services in the DOTS clinic, proper counseling, health education, and appropriate follow up by the service providers and presence of comorbidities.

The HIV coinfection prevalence among TB patients found in this study was 8.3%. This finding is higher than global current average prevalence 6.7%, but lower than that of WHO African region 19.7% of HIV-positive cases among all TB cases.^[29]^ The global TB report of 2022 also displayed that Ethiopia national current average of HIV coinfection among all TB cases is 5.2%^[30]^ which is lower than the prevalence observed in our study. The finding observed in this study indicated that why Ethiopia is considered to continue as 1 of the high burden TB/HIV coinfection countries globally.^[29]^ This prevalence reveals that the HIV infection across the community of the study areas is high, which is characterized by HIV prevalence of greater than or equal to 5% among TB patients.^[31]^ This finding is lower than that of Woldia General Hospital in Northeast Ethiopia,^[21]^ Eastern Ethiopia,^[13]^ Southern Ethiopia,^[19]^ but similar with that of Bule Hora General Hospital in Southern Ethiopia,^[14]^ Western Ethiopia,^[18]^ Ghana^[28]^ and Arsi Southern Ethiopia.^[17]^ These variations might be due to differences in level of town, HIV prevalence in the community, study setting and number of HIV coinfection out of ART associated TB.

HIV status influenced TB treatment outcomes observed in this study. Moreover, HIV-positive patients have a higher risk of experiencing unsuccessful treatment outcome compared to HIV-negative TB patients. The unsuccessful treatment outcome of all TB recorded in this study was 9% which comprised of 20% of HIV-positive and 8.1% of HIV-negative patients. Unsuccessful treatment outcome associated with HIV-positive TB patients have been reported by numerous studies: from Addis Ababa Ethiopia,^[10]^ Northwest Ethiopia,^[32]^ Eastern Ethiopia,^[22]^ Ghana,^[16]^ and Malawi.^[33]^ The reasons for the unsuccessful treatment outcome may be due to inadequate support provided to people with TB/HIV coinfection to ensure high level of adherence,^[34]^ late diagnosis of HIV,^[35]^ pill burden and drugs interactions.^[33]^ Strong tracing mechanism and TB/HIV collaborative activities required to improve TB treatment outcomes, especially for HIV-positive patients, and establishing active feedback system with the health facilities to which patients transferred out required to improve the treatment outcome of TB.

Globally, the number of TB incident cases have been falling slowly since 2000^[36]^; however, in this study, the number of TB cases increased gradually from 84 cases in 2017/8 to 92 cases in 2021/22 with a peaked rate at the year 2020/21 which showed the rate is growing in general across the community in the study areas. This increment was due to HIV-positive cases with a sharp increase in 2020/21 although TB/HIV coinfection rate have been falling since 2008 worldwide^[37]^ and increasing rate in HIV-negative cases from 76 cases in 2017/8 to 85 cases in 2021/22 with a sharp rise in 2020/21 recorded in this study. This finding is consistent with global estimates of high incident cases expected in 2021 and beyond because of the negative impact of the COVID-19 pandemic on TB diagnosis and treatment and on broader TB determinants, such as income levels and undernutrition, which increase the probability of developing TB disease among people already infected with mycobacterium TB.^[38]^ This gradual increase in TB cases in our study suggests additional efforts to strengthen the TB/HIV collaborative activities and set of measures to TB infection control at health facility level can better reduce this increasing volume of TB cases which enable us to achieve the End TB targets.

In this study, age showed statistically significant association with TB treatment outcome. It was observed that TB patients within the age groups 20 to 35 years old were more likely to have successful treatment outcome compared the patients older than 35 years old. This findings were consistent with the report from other studies from Southeast Ethiopia,^[39]^ Eastern Ethiopia,^[13]^ Northeast Ethiopia,^[21]^ Southeast Ethiopia,^[40]^ Ghana^[16]^ and Central Ethiopia.^[12]^ On the other hand, the findings of our study were not in agreement with several studies reported no association of age with treatment outcome from Debre Tabor General Hospital in Northwest Ethiopia,^[26]^ Jinka,^[19]^ and Southern Ethiopia.^[17]^

The data of this study showed statistically significant association between residence and TB treatment outcome, and revealed that urban dwellers had more chances of having successful treatment outcome than those who resided in rural areas. The finding of this study was in agreement with the studies reported from Western Ethiopia,^[18]^ and Northwest Ethiopia,^[32]^ but not in line with the study elsewhere that showed no association between residence and treatment outcome of TB patients in Southern Ethiopia,^[25]^ and Northeast Ethiopia.^[21]^ The finding in our study might be due to the high transfer out patients whose treatment outcomes were not reported back by rural health centers the patients transferred out to and not recorded in the hospital. The other reasons reported by other study could be applicable to our finding such as higher awareness and information about TB, HIV and their treatments, and less distance to health facility urban residents have might contribute to the higher rate of successful treatment outcome in patents came from urban areas.^[18]^

## 5. Conclusion

### 5.1. Demographic and clinical characteristics

The study included a total of 420 TB patients, with a median age of 22 years. The majority of both HIV-positive (74.3%) and HIV-negative (82.9%) TB patients were younger than 36 years. There were no significant differences between the 2 groups in terms of year of treatment, age, sex, place of residence, and pretreatment weight.

### 5.2. Tuberculosis cases by human immunodeficiency virus status

Among the participants, 8.3% were TB-HIV coinfected patients. The proportion of HIV-positive TB cases showed a decreasing trend from 2017/8 to 2019/20, followed by a sharp rise in 2020/21 and a slight decrease in the most recent period. Overall, there was an increasing trend in TB cases, but the rate was smaller among HIV-positive cases compared to HIV-negative cases, although the difference was not statistically significant.

### 5.3. Disease characteristics

The majority of patients (95.5%) had no previous history of TB treatment, indicating a high number of newly diagnosed cases. Approximately 40.2% of both HIV-positive and HIV-negative TB patients had smear-positive pulmonary TB. There was a statistically significant difference in treatment duration between HIV coinfected and HIV-negative TB patients. However, there were no significant differences in previous history of TB treatment, TB treatment category, TB type, and DM between HIV-negative and HIV-positive TB patients.

### 5.4. Treatment outcomes

The overall treatment success rate for all TB patients was 91.0%. The success rate for HIV-positive TB patients was 80.0%, while it was 91.9% for HIV-negative TB patients. HIV-positive patients had a higher rate of unsuccessful treatment outcomes (20%) compared to HIV-negative patients (8.1%). HIV-positive TB patients were more than twice as likely to die and twenty-two times more likely to be lost to follow-up than HIV-negative TB patients.

### 5.5. Factors associated with treatment outcome

In multivariable analysis, HIV status, age, and residence were significantly associated with TB treatment outcomes. HIV-negative TB patients were 2.561 times more likely to have a successful treatment outcome compared to HIV-positive patients. The odds of successful treatment outcomes were 2.95 times higher in the age group of 20 to 35 years compared to those above 35 years. Patients residing in urban areas had 2.961 times higher odds of successful treatment outcomes compared to rural dwellers.

## Acknowledgments

We acknowledged Haramaya University Colleges of Health and Medical Sciences Institutional Health Research Ethical Review Committee for giving the ethical clearance. We also thank all individuals who have in one way or another contributed to the completion of this study.

## Author contributions

**Conceptualization:** Fikru Tebeje, Adnan Ahmed, Fitsum Weldegebreal, Yadeta Dessie.

**Data curation:** Fikru Tebeje, Adnan Ahmed, Fitsum Weldegebreal, Yadeta Dessie.

**Formal analysis:** Fikru Tebeje, Adnan Ahmed, Fitsum Weldegebreal, Yadeta Dessie.

**Funding acquisition:** Fikru Tebeje.

**Investigation:** Fikru Tebeje, Adnan Ahmed, Fitsum Weldegebreal, Yadeta Dessie.

**Methodology:** Fikru Tebeje, Adnan Ahmed, Fitsum Weldegebreal, Yadeta Dessie.

**Project administration:** Fitsum Weldegebreal.

**Software:** Fikru Tebeje, Adnan Ahmed, Fitsum Weldegebreal, Yadeta Dessie.

**Supervision:** Fikru Tebeje, Fitsum Weldegebreal, Yadeta Dessie.

**Validation:** Fikru Tebeje, Adnan Ahmed, Fitsum Weldegebreal, Yadeta Dessie.

**Visualization:** Fikru Tebeje, Adnan Ahmed, Fitsum Weldegebreal, Yadeta Dessie.

**Writing – original draft:** Fikru Tebeje, Adnan Ahmed, Fitsum Weldegebreal, Yadeta Dessie.

**Writing – review & editing:** Fikru Tebeje, Adnan Ahmed, Fitsum Weldegebreal, Yadeta Dessie.
